# NEUROPSYCHIATRIC SYMPTOMS AND MORTALITY FOR COGNITIVELY NORMAL OLDER MEXICAN AMERICANS

**DOI:** 10.1093/geroni/igac059.2086

**Published:** 2022-12-20

**Authors:** Phillip Cantu, Soham Al Snih, Kyriakos Markides

**Affiliations:** The University of Texas Medical Branch, Galveston, Texas, United States; UTMB, Galveston, Texas, United States; UTMB, Galveston, Texas, United States

## Abstract

**Objectives:**

Neuropsychiatric symptoms (NPS) present in older adults with Alzheimer’s disease (AD) and other dementias are related to mortality. Research on the relationship between NPS and mortality in a non-dementia population has not been conducted. This study examines NPS as a predictor of six-year mortality among community dwelling Mexican Americans aged 80 years and older.

**Methods:**

Data included 466 cognitively normal participants from wave 7 of the Hispanic Established Population for the Epidemiologic Study of Elderly. NPS were measured using the Neuropsychiatric inventory (NPI). Cox proportional hazard modes were used to estimate the hazard ratio (HR) of mortality.

**Results:**

The hazard ratio death at six years was 1.02 (95 % CI, 1.00-1.04) as a function of having any NPI score and 1.09 (95% CI 1.02-1.17) for number of NPI conditions, controlling for demographic and health characteristics. Apathy, irritability, and aberrant motor behavior were all independently predictors of mortality.

**Conclusions:**

NPS may be modifiable risk factors to increase survival time or may by indicative of underlying healthy problems. NPS may be related to underlying health conditions among older adults with normal cognitive functioning.

